# Reliability and validity of the modified child and adolescent physical activity and nutrition survey (CAPANS-C) questionnaire examining potential correlates of physical activity participation among Chinese-Australian youth

**DOI:** 10.1186/1471-2458-14-145

**Published:** 2014-02-11

**Authors:** Claudia Strugnell, Andre Renzaho, Kate Ridley, Cate Burns

**Affiliations:** 1 School of Exercise and Nutrition Sciences, Deakin University, 221 Burwood Hwy, Burwood, Victoria 3125, Australia; 2 International Public Health Unit, Department of Epidemiology & Preventative Medicine, Monash University, Level 3 Burnet Building, 89 Commercial Road, Melbourne, Victoria 3004, Australia; 3 Centre for Sport, Health and Physical Education (SHAPE), School of Education, Flinders University, GPO Box 2100, Adelaide, South Australia 5001, Australia; 4 School of Dentistry and Health Services, Charles Sturt University, Wagga Wagga 2678, Australia

**Keywords:** Physical activity, Reliability, Adolescents, Chinese-Australians

## Abstract

**Background:**

To date, few questionnaires examining psychosocial influences of physical activity (PA) participation have been psychometrically tested among Culturally and Linguistically Diverse (CALD) youth. An understanding of these influences may help explain the observed differences in PA among CALD youth. Therefore, this study examined the reliability and predictive validity of a brief self-report questionnaire examining potential psychological and social correlates of physical activity among a sample of Chinese-Australian youth.

**Methods:**

Two Chinese-weekend cultural schools from eastern metropolitan Melbourne consented to participate in this study. In total, 505 students aged 11 to 16 years were eligible for inclusion in the present study, and of these, 106 students agreed to participate (21% response rate). Participants completed at 37-item self-report questionnaire examining perceived psychological and social influences on physical activity participation twice, with a test–retest interval of 7 days. Predictive validity, internal consistency and test–retest reliability were evaluated using exploratory factor analyses, Cronbach’s α coefficient, and the intraclass correlation coefficient (ICC) respectively. Predictive validity was assessed by correlating responses against duration spent in self-reported moderate-to-vigorous physical activity (MVPA).

**Results:**

The exploratory factor analysis revealed a nine factor structure, with the majority of factors exhibiting high internal consistency (α ≥ 0.6). In addition, four of the nine factors had an ICC ≥ 0.6. Spearman rank-order correlations coefficients between the nine factors and self-reported minutes spent in MVPA ranged from -0.5 to 0.3 for all participants.

**Conclusion:**

This is the first study to examine the psychometric properties of a potential psychological and social correlates questionnaire among Chinese-Australian youth. The questionnaire was found to provide reliable estimates on a range of psychological and social influences on physical activity and evidence of predictive validity on a limited number of factors. More research is required to improve the reliability and validity of the questionnaire.

## Background

Regular physical activity (PA) participation has many documented health benefits for children and adolescents [1]. To guide intervention design and enhance current understanding of PA, several ecological models have been developed for children, adolescents and adults [2]. These models provide a framework for understanding the complex array of influences on PA and highlight that no singular influence or correlate is responsible for PA, rather, a combination of different types of influences (e.g. policy, environmental, social and psychological) are involved.

Studies examining the correlates or influences of PA among youth are abundant, with several reviews using an ecological approach to synthesise the available evidence [3-5]. Several studies within China have investigated these influences among Chinese youth [6-9], however, investigations among youth from an Asian Culturally and Linguistically Diverse (CALD) background outside of Asia are less apparent [10-12]. Investigations among Asian youth outside of Asia are needed as their PA levels have consistently been shown to be lower than their non-CALD peers (predominantly from a European background) [13-20]. Additionally, correlates of PA among Asian CALD youth have been shown to differ from their non-CALD peers in various regions and/or counties outside of Asia [10-12]. Recent investigations comparing Vietnamese-Australian and Anglo-Australian adolescents (aged 12-14 years) have shown considerable differences in perceptions and correlates of PA participation [10,11]. For example, Wilson & Dollman [10] found maternal encouragement for PA significantly predicted minutes spent in moderate-to-vigorous physical activity (MVPA) among Anglo-Australian female adolescents but not Vietnamese-Australian females, for which maternal co-participation was important [11]. In contrast, a separate study among Vietnamese-Australian males found no significant associations with MVPA participation among Anglo-Australian males, whereas fathers’ encouragement and support for PA predicted MVPA participation among Vietnamese-Australian males [10]. Given that the influences on PA are likely to vary widely among differing populations, population subgroups [21], cultures and gender subgroups; population-specific studies are needed with CALD-appropriate instruments. In addition, CALD differences in perceived influences on PA may relate to the appropriateness and accuracy of instruments in examining related constructs. Therefore, the aim of the current study was to investigate the test-retest reliability, internal consistency and predictive validity of a modified questionnaire among Chinese-Australian youth aged 11–14 years which is intended to be used in a subsequent cross-sectional study among Chinese-Australian youth in metropolitan Melbourne. The original questionnaire (Child and Adolescent Physical Activity and Nutrition Survey, for Years 8, 10 and 11) has previously been used among Australian children and adolescents [22,23], although this article reports on the modified questionnaire items which related to perceived psychological and social influences on PA.

## Methods

Three Chinese-weekend cultural schools from eastern metropolitan Melbourne were invited into the study. Two schools comprising of three separate school campuses consented to participate. As all Chinese-weekend cultural schools are grouped into classes based on language ability rather than age, all students in language levels 7–8 whose age ranged between 11 and 16 years were invited to participate (N = 505). Informed written consent was obtained from the student’s parent/guardian and the student provided assent. In total, 106 students returned a signed consent form and completed the initial questionnaire during class time (response rate of 21%). Ethical approval was sought and granted by the Faculty of Health Human Ethics Advisory Group of Deakin University and the Victorian Department of Education and Early Childhood Development prior to conducting this study.

All twenty-five items from the original Child and Adolescent Physical Activity and Nutrition Survey (CAPANS) questionnaire for Years 8, 10 and 11 students that examined perceived influences on PA were included in the modified potential psychological and social correlates of PA instrument (CAPANS-C) [23]. These items were used with permission from the developer; the Evaluation and Monitoring Working Group of the Western Australian Premier’s Physical Activity Taskforce. Although, most items within the original CAPANS questionnaire derived from the Children’s Leisure Activities Study Survey [24] and the 1997 NSW Schools Fitness and Physical Activity Survey [25]. Among Australian school-children (aged 10–12 years), several previous versions of these question items have been reliability tested with consistency in subjects’ responses to PA questions over a 7-day retest period producing kappa indices of agreement ranging from 0.30-0.58 [24]. In the current study, twelve new psychological or social items were developed for the CAPANS-C instrument based on their hypothesized relationship with PA using an ecological approach [2] and previous research findings [10,11,26]. Eleven of the new items were hypothesized to be negatively associated with PA participation and included: ‘None of my friends want to do more exercise or activity with me’, ‘Mum/Dad don’t encourage me to be physically active’, ‘Mum/Dad don’t help me be physically active’, ‘Mum/Dad isn’t physically active with me’, ‘Mum/Dad thinks education/study is more important than physical activity’ and being physically active over the next year might ‘Prevent me from doing homework/study’; whilst a positive association was hypothesized for ‘Mum/Dad does a lot of physical activity’ and PA participation. These items were developed based on the observed differences in parental support for PA among Vietnamese-Australian adolescents [10,11] in addition to the finding that parents of Asian background students in Australia are influential in the student’s motivation for academic performance [26]. In total, the CAPANS-C questionnaire comprised 37 statements which may encourage or inhibit PA participation and responses were provided on a 6-point Likert scale [Strongly Agree (1), Agree (2), Neither (3), Disagree (4), Strongly Disagree (5) and Don’t Know (6)]. The acceptability, readability and subsequent content validity of the newly developed and existing question items were approved by the study’s steering committee which comprised of the listed authors (one of whom is a Primary and Secondary school teacher), Dr Jisheng Cui and a representative from the Australian Chinese Medical Association (Victoria) Inc.

Participants also completed a self-report PA and sedentary behaviour questionnaire which has been psychometrically tested among Chinese-Australian youth aged 11–14 years (CAPANS-PA) [27]. Among Chinese-Australian youth, test-retest reliability of self-reported duration spent in total MVPA (Mon-Sun) (mins.wk^-1^) was high [Intraclass correlation (ICC) = 0.78, P < 0.01] [27], although the evaluation of the construct validity against accelerometry measured duration spent in weekly Mon-Sun MVPA (min.d^-1^) showed poor and statistically non-significant correlation (ρ = 0.07) [28]. The questionnaire also included several demographic questions [age, gender, suburb and postcode of residence and country of birth of the participant, their biological mother and father and their maternal grandmother and grandfather].

Both questionnaires were conducted in English during May and August 2009, which took approximately 15 minutes of class time at Time 1 (T1), with 77 participants completing the questionnaire again, 7-days later at Time 2 (T2). The first author or trained data collectors guided participants through both questionnaires and each student received compensation in the form of a recyclable bag containing a ‘hacky sack’ and three Australian Government brochures pertaining to healthy eating and regular PA.

Data analyses were conducted using SPSS Statistics Version 17.0. Twelve items were reversely coded so that a higher value indicated a positive effect on PA participation and are indicated in Table 1 and Table 2. Responses to the 37 items were submitted to two factor analytic procedures (due to differing questionnaire stems) to identify any sub-scales using principal component extraction method with oblimin rotation and Kaiser normalisation [29]. The first 25 items were submitted to Component One as the question stem asked “How much do you agree with the following things?” and the remaining 12 items to Component Two factor analyses as the question stem asked “How much do you agree with the following things? Being physically active over the next year might…”. The Kaiser-Meyer-Olkin (KMO) value was used to measure the sampling adequacy and a cut-off point of ≥ 0.6 was implemented for a satisfactory factor analysis to proceed (Component One KMO = 0.63; Component Two KMO = 0.69) [30]. The strength of the variables was also tested using the Bartlett's test of sphericity to reject the null hypothesis that the correlations in the correlation matrix are zero at P < 0.05 [30]. Bartlett's test of sphericity returned a Chi-Square of 912.2, P < 0.001 for Component One and a Chi-Square of 295.7, P < 0.001 for Component Two. For a factor to be retained, three conditions had to be met: 1) a clear structure after rotation; 2) contained two or more items with a factor loading > 0.35; [30] and 3) an initial Eigen value ≥1.0. The scree plot and initial Eigen values were used to help determine the number of factors to retain in the component. A subsequent factor analysis was conducted forcing the correct number of factors (identified in the scree plot) to make further assessments of fit. Unweighted and weighted scores were computed for each factor. Unweighted scores were calculated by adding each item’s raw score within a factor. Weighted scores were generating using the method described by De Vaus [31], whereby each individual item is multiplied by the corresponding item loading within the factor [31]. This weighting enables the contribution of each item to the overall factor to be reflected.

**Table 1:** Exploratory factor analysis results for component one (Items 1–25)

**Item**	**Factor**					
**“How much do you agree with the following things?”**	**Social- parental support**	**Psychological-perceived barriers**	**Psychological- preference/attitude**	**Psychological- perceived ailment**	**Social-family education beliefs**	**Social – parental modelling**
Mum doesn’t encourage me to be PA	.661					
Dad doesn’t encourage me to be PA	.795					
Dad doesn’t help me be PA	.691					
Mum doesn’t help me be PA	.589					
Mum isn’t physically active with me	.659					
Dad isn’t physically active with me	.795					
I don’t have proper equipment to play sport		.754				
I don't have anyone to be PA with		.617				
I don’t like how being PA makes me feel		.568				
There are no parks/sporting grounds near where I live		.410				
I don’t like physical activity			.646			
I don’t think I am very good at PA			.585			
I prefer to watch TV or play electronic games			.558			
I look funny when I am physical activity			.508			
I am scared that I might get hurt if I played a sport			.464			
I have an injury that prevents me being PA				.783		
I have a health problem				.775		
Others laugh at me when I am PA				.681		
Dad thinks education is more important than PA					.848	
Mum thinks education is more important than PA					.791	
Dad does a lot of physical activity^⌂^						.834
Mum does a lot of physical activity^⌂^						.710
Eigen value	5.1	2.3	1.9	1.8	1.7	1.4
Cronbach's alpha (α)	0.8	0.6	0.6	0.7	0.8	0.6
Percent variance explained (%)	20.2	9.4	7.2	7.2	6.9	5.8
Weighted factor based scale score^*a*^ (median & IQR)	16.8 (14.5, 19.4)	9.9 (8.9, 11.1)	11.2 (10.0, 12.6)	11.2 (9.6, 11.2)	3.3 (3.3, 5.8)	4.7 (3.9, 6.9)
Unweighted factor based scale score^*b*^ (median & IQR)	24.5 (21.0, 28.0)	17.0 (15.0, 19.0)	20.0 (18.0, 23.0)	15.0 (13.0, 15.0)	4.0 (4.0, 7.0)	6.0 (5.0, 9.0)

Note: Principal Component extraction method with Oblim rotation (Kaiser Normalisation); ^*a*^ = weighted factor based scale score using the method described by de Vaus; ^*b*^ = Unweighted factor based scale score mean; and PA = physically active or physical activity and ^⌂^ = Item has been reversely coded so that a higher score is positively linked with physical activity participation.

**Table 2:** Exploratory factor analysis results for component two (Items 26–37)

**Item**	**Factor**		
**“How much do you agree with the following things? Being physically active over the next year might:”**	**Psychological- personal benefits**	**Psychological- positive outcome expectations**	**Psychological- negative outcome expectations**
Keep me healthy^⌂^	.673		
Improve my appearance^⌂^	.515		
Make me feel good about myself^⌂^	.703		
Make or keep me fit^⌂^	.745		
Help me lose weight or help me control my weight^⌂^	.449		
Let me have a lot of fun^⌂^	.673		
Help me study and learn better^⌂^		.666	
Make my parents/carers happy^⌂^		.509	
Help me spend time with my friends^⌂^		.747	
Help me make new friends^⌂^		.825	
Prevent me from doing homework/study			.755
Prevent me doing other things I like more			.716
Eigen value	3.4	1.5	1.3
Cronbach's alpha (α)	0.7	0.7	0.5
Percent variance explained (%)	28.5	12.1	11.0
Weighted factor based scale score^*a*^ (median & IQR)	16.7 (15.1, 17.8)	10.5 (9.2, 13.0)	4.4 (3.6, 5.2)
Unweighted factor based scale score^*b*^ (median & IQR)	26.0 (24.0, 28.0)	15.0 (13.0, 19.0)	6.0 (5.0, 7.0)

Note: Principal Component extraction method with oblim rotation (Kaiser Normalisation); ^*a*^ = weighted factor based scale score using the method described by de Vaus; ^*b*^ = Unweighted factor based scale score mean; and ^⌂^ = Item has been reversely coded so that a higher score is positively linked with physical activity participation.

The internal consistency of each factor was assessed using the acceptable Cronbach’s alpha value of α ≥0.6 [32]. Predictive validity was examined using Spearman rank-order correlations coefficients (Rho, ρ) between each factor score and duration spent in total MVPA (Mon-Sun) (mins.wk^-1^). Paired-sample Wilcoxon signed-rank tests were used to examine intra-individual differences in median responses from T1 to T2. The test-retest reliability of the CAPANS-C questionnaire was assessed among 77 participants who completed the questionnaire twice, within 7-days. An ICC ≥ 0.7 was considered evidence of acceptable reliability [33]. Predictive validity and test-retest reliability was examined for both boys and girls and the entire sample.

## Results

The 105 students with complete T1 information were aged between 11–14 years (mean ± SD: 12 ± 0.8 years) with an almost equal distribution of boys (44%) and girls (56%) participating. Eighty-three percent participants were of Chinese ethnicity by either being born in China themselves (8%) or having both parents born in China or having both maternal grandparents being born in China (excluding: Taiwan, Hong Kong and Macau) (75%). The remaining 17% were predominantly from a South-East Asian background (e.g. Malaysia, Vietnam and Cambodia).

The results of the exploratory factor analysis for Component One of the CAPANS-C are presented in Table 1. A total of six factors were identified and were named based on the predominant level of influence using an ecological model (psychological or social) and the items within each factor. Three items were removed during analyses. The item ‘I do a lot of physical activity’ was excluded as it considerably reduced the alpha coefficient for Factor 3 ‘Psychological preference/attitude’ (α = 0.5 included, α = 0.6 excluded). Additionally, two items consisting of ‘None of my friends want to do more exercise or activity with me’ and ‘I don’t have enough time for physical activity’ were excluded as they did not achieve a factor loading >0.35 across any of the six factors. In general, acceptable internal consistency was evident for all factors [34].

Results of the Component Two analyses revealed a 3 factor structure which is presented in Table 2. All but one factor had acceptable internal consistency, with the ‘Negative outcome expectation’ factor having slightly lower than desired internal consistency (α = 0.5) [32]. Test-retest reliability and median factor scores from the 77 participants with complete Time 1 and Time 2 data are presented in Table 3. Intra-class correlation coefficients ranged from 0.3 to 0.8, with two factors have acceptable test-retest reliability overall ‘Parental modelling’ and ‘Personal benefits’. Gender differences in reliability were observed with boys (only) having acceptable reliability for two factors ‘Parental support’ and ‘Perceived barriers’ with girls (only) for the ‘Family education beliefs’ factor. When examining intra-individual variation between T1 and T2 administration, significant variation was only evident for the ‘Negative outcome expectations’ factor overall (P < 0.05).

**Table 3:** **Test-retest reliability of factors and correlations with self-reported MVPA (mins.wk**^**-1**^**)**

	**N**	**Median**^**a **^**(IQR)**		**ICC**^**b**^	**N**	**Correlation**
		**Time 1 T1**	**Time 2 T2**	**(95%: CI)**		**(Rho, ρ)**
**Social – parental support**						
**Overall**	77	24.0 (21.0, 28.0)	24.0 (22.0, 29.0)	0.6 (0.40, 0.87)	104	0.24* Weighted
						0.25* Unweighted
**Boys**	38	26.5 (23.0, 29.0)	25.5 (24.0, 30.0)	0.8 (0.55, 0.98)	45	0.18 Weighted
						0.21 Unweighted
**Girls**	39	24.0 (20.0, 26.0)	24.0 (18.0, 26.0)	0.5 (0.08, 0.83)	52	0.16 Weighted
						0.16 Unweighted
**Psychological – perceived barriers**						
**Overall**	77	17.0 (15.0, 19.0)	16.0 (15.0, 19.0)	0.6 (035, 0.90)	99	0.24* Weighted
						0.26** Unweighted
**Boys**	38	18.0 (16.0, 19.0)	18.0 (16.0, 19.0)	0.7 (0.38, 0.99)	46	0.31* Weighted
						0.33** Unweighted
**Girls**	39	16.0 (14.0, 18.0)	16.0 (14.0, 18.0)	0.6 (0.22, 0.90)	54	0.16 Weighted
						0.19 Unweighted
**Psychological – preference/attitude**						
**Overall**	77	21.0 (18.0, 23.0)	20.0 (18.0, 23.0)	0.5 (0.25, 0.78)	98	0.27** Weighted
						0.24* Unweighted
**Boys**	38	21.0 (19.0, 23.0)	20.5 (18.0, 24.0)	0.4 (0.05, 0.79)	46	0.25 Weighted
						0.23 Unweighted
**Girls**	39	20.0 (18.0, 23.0)	20.0 (18.0, 22.0)	0.6 (0.25, 0.89)	52	0.26 Weighted
						0.24 Unweighted
**Psychological- perceived ailment**						
**Overall**	76	15.0 (13.0, 15.0)	15.0 (12.0, 15.0)	0.5 (0.14, 0. 94)	98	-0.11 Weighted
						-0.11 Unweighted
**Boys**	37	15.0 (12.0, 15.0)	14.0 (12.0, 15.0)	0.4 (0.00, 0.87)	45	0.07 Weighted
						0.09 Unweighted
**Girls**	39	15.0 (13.0, 15.0)	15.0 (12.0, 15.0)	0.6 (0.20, 1.04)	54	-0.18 Weighted
						-0.20 Unweighted
**Social- family education beliefs**						
**Overall**	76	4.0 (4.0, 6.0)	5.0 (4.0, 6.0)	0.3 (0.00, 0.62)	96	0.12 Weighted
						0.12 Unweighted
**Boys**	38	4.5 (4.0, .0)	5.0 (4.0, 6.0)	0.4 (0.04, 0.81	45	0.00 Weighted
						0.00 Unweighted
**Girls**	38	4.0 (4.0, 6.0)	5.0 (4.0, 6.0)	0.7 (0.45, 0.81)	51	0.26 Weighted
						0.26 Unweighted
**Social – parental modelling**						
**Overall**	76	6.5 (5.0, 8.5)	6.0 (4.5, 8.0)	0.7 (0.41, 0.95)	97	-0.26** Weighted
						-0.26* Unweighted
**Boys**	38	6.0 (5.0, 8.0)	6.0 (5.0, 8.0)	0.7 (0.42, 0.99)	46	-0.47** Weighted
						-0.45** Unweighted
**Girls**	38	7.0 (6.0, 9.0)	6.0 (4.0, 8.0)	0.7 (0.40, 0.97)	51	-0.08 Weighted
						-0.08 Unweighted
**Psychological – personal benefits**						
**Overall**	75	26.0 (24.0, 28.0)	26.0 (24.0, 29.0)	0.7 (0.42, 0.92)	97	0.16 Weighted
						0.14 Unweighted
**Boys**	36	26.0 (24.0, 27.5)	26.0 (23.5, 28.5)	0.7 (0.39, 0.94)	44	0.01 Weighted
						0.01 Unweighted
**Girls**	39	25.0 (24.0, 29.0)	26.0 (24.0, 30.0)	0.7 (0.39, 0.96	53	0.26 Weighted
						0.24 Unweighted
**Psychological- positive outcome expectations**						
**Overall**	77	16.0 (14.0, 19.0)	15.0 (14.0, 18.0)	0.4 (0.07, 0.66)	99	0.17 Weighted
						0.17 Unweighted
**Boys**	38	15.0 (14.0, 18.0)	15.0 (14.0, 18.0)	0.3 (0.00, 0.72)	46	-0.01 Weighted
						-0.03 Unweighted
**Girls**	39	16.0 (13.0, 19.0)	15.0 (14.0, 18.0)	0.4 (0.00, 0.72)	53	0.30* Weighted
						0.30* Unweighted
**Psychological- negative outcome expectations**						
**Overall**	73	6.0 (5.0, 7.0)	6.0 (5.0, 9.0)*	0.4 (0.07, 0.71)	99	-0.13 Weighted
						-0.14 Unweighted
**Boys**	38	6.0 (5.0, 7.0)	6.0 (5.0, 9.0)	0.4 (0.00, 0.76)	46	-0.47*** Weighted
						-0.47*** Unweighted
**Girls**	39	6.0 (4.0, 7.0)	6.0 (6.0, 9.0)	0.6 (0.21, 0.93)	53	0.07 Weighted
						0.08 Unweighted

^a^= median and IQR; ^b^= Intra-class correlation and 95% confidence interval based on unweighted factor scores; ρ = Spearman rank-order correlation based on unweighted and weighted factor score; with significant correlations (in bold) indicated as follows, *P = <0.05, **= P<0.01, ***P = <0.001; and N = number of respondents for the question item.

Table 3 also presents the predictive validity results (Spearman rank-order correlation coefficients) between each of the eight factors and self-reported duration spent in MVPA (mins.wk^-1^) using available T1 data from the 105 participants. Significant positive correlations between MVPA were observed for the ‘Parental support, ‘Perceived barriers’, and the ‘Preference/attitude’ factors overall (P < 0.05). Gender differences in predictive validity were also evident with girls (only) having a significant positive correlation with the ‘positive outcome expectation’ factor and boys (only) having a significant negative correlation with the ‘Negative outcomes expectation’ factor (P < 0.05).

## Discussion

This study is believed to be the first to examine the psychometric properties of a potential psychological and social correlates of PA questionnaire among Chinese-Australian youth. The results indicate the instrument provided stable and valid estimates for several psychological and social factors relating to PA participation in this population. The CAPANS-C instrument may prove to be useful when examining psychological and social influences on PA among similar Asian-Australian populations.

In this study, a nine factor structure was supported by the exploratory factor analysis with all but one factor having acceptable internal consistency [32]. Test-retest reliability of the nine factors was shown to be acceptable for 2/9 factors for all participants, 4/9 for boys and 3/9 for girls if using the criteria of an ICC ≥ 0.7 [33]. However, lower cut-points for acceptable reliability have been applied in similar studies with acceptable reliability defined as ICC ≥ 0.6 [32,34]. If the lower criteria were applied, the questionnaire would have acceptable reliability for 4/9 factors for boys and all participants, and for 6/9 factors among girls, further supporting the stability of the questionnaire.

Gender differences in predictive validity were evident, with boys having a higher number of significant correlations with MVPA (3/9 factors) compared to girls (1/9 factors). In particular, the ‘Perceived barriers’ and ‘Parental modelling’ factors differed between genders with boys (only) having evidence of predictive validity and acceptable test-retest reliability. These findings suggest poor stability of these factors among Chinese-Australian girls as no significant intra-individual variations were observed. Interestingly, the parental modelling factor was negatively associated with MVPA participation among boys (r = -0.47, P < 0.01), which is in contrast to what was hypothesised (positive relationship). Due to the recoded nature of these newly developed items, boys who perceived their parents to engage in more physical activity engaged in less MVPA. In the wider literature, there is insufficient evidence for an association between parental physical activity levels and adolescents’ physical activity [3,4]. Suggesting that this finding is a CALD-specific association among Chinese-Australian boys. Among Vietnamese-Australian boys and girls, parental modelling was not associated with adolescent's MVPA participation [10,11]. Similar to the current study, perceived paternal help towards physical activity was significantly associated with MVPA among Vietnamese-Australian boys (r = 0.23, p < 0.05) [10], but not girls [11]. The nature of parental modelling and offspring's participation in MVPA requires further investigation among Chinese-Australian youth and should be investigated for gender and parent type (mother or father) differences within this population.

Whilst direct comparisons with the present findings to previous research are inappropriate, findings from similar studies among Asian youths within Asia are mixed [35,36]. In a perceived psychological influences (self-efficacy, benefits and barriers) of PA questionnaire validation among Taiwanese adolescents, a three factor structure was evident for the perceived benefits (health, appearance and psychosocial function) and perceived barriers (time constraints, lack of facilities, and personal issues) [36]. In addition, all factors were significantly correlated with PA (r = -0.19 to 0.47) and had acceptable internal consistency. These findings are similar to the present study, where a ‘personal benefits’ and ‘perceived barriers’ factor emerged and related to perceived health/appearance benefits and perceived personal barrier concepts, however, only the ‘perceived barriers’ factor was significantly associated with MVPA duration in this study. Large methodological differences between the two studies are likely to explain these observed differences (e.g. study design, sample size, question statements and stems, scoring options and population-specific differences). In particular, the description of how PA duration was measured among Taiwanese adolescents was not included [36], and therefore may be objectively measured or self-reported PA and examine all PA intensities, not MVPA specifically.

Another pertinent questionnaire validation among Hong Kong Chinese youth (9-14y) examined the reliability and validity properties of a potential psychological, social and environmental correlates questionnaire [35]. Among Hong Kong Chinese youth, peer support for PA was positively associated with self-reported MVPA duration (r = 0.25, P < 0.05) and family support was negatively associated with screen time (r = -0.22, P < 0.05) [35+], with both factors having acceptable test-retest reliability. In the current study, the ‘Parental support’ factor had acceptable test-retest reliability overall if using the lower criterion and was positively associated with MVPA (r = 0.24 P < 0.05), similarly confirming the rationale for the development of the 4 new items within this factor in relation to predictive validity of support constructs for physical activity participation.

The development of two new items to investigate perceived parental educational beliefs among Chinese-Australian youths was of particular interest in this study. These two items ‘Mum/Dad thinks education/study is more important than physical activity’ were created in response to findings that parents of Asian adolescents in Australia are a significant impetus to achieve academically [26]. It was hypothesized that a negative relationship with physical activity be observed, that is that adolescents who perceived that their parents viewed education as more important than physical activity engage in less physical activity. In the factor analyses, these two items loaded across the singular ‘family education beliefs’ factor and had acceptable internal consistency but did not have acceptable test-retest reliability or evidence of predictive validity. As these items were specifically created for application in the current study, more research is required to develop a robust item(s) that measures perceived parental academic pressure among Chinese-Australian youths. Additionally, it is recommended the predictive validity of this item on sedentary behaviour participation be examined..

The strengths and limitations of this study must be acknowledged. The primary strength relates to the successful recruitment and engagement of Chinese-Australian youth specifically in multicultural Melbourne, Australia and the psychometric testing of a perceived correlates of PA questionnaire. A recent review of instruments used to examine potential mediators of PA found few studies reported validity properties and these were often low when reported [34]. Therefore, the current study contributes to the paucity of psychometrically tested instruments, in addition to being among a CALD population. With further reliability and validity testing, this instrument may be an important tool for future investigations and interventions among Asian youth in Australia and internationally.

Pertinent limitations relate to the small sample size used in the exploratory factor analysis, which is likely to have influenced the observed correlation matrix. Various specifications for generating sample sizes for factor analyses have been proposed including a general rule of a minimum number of participants (e.g. 300), specifying a minimum N as a function of the number of variables (e.g. a ratio of 4 subjects: 1 variable) or specifying a finite number [37]. However, it has been demonstrated that if the communalities of a factor (amount of variance accounted for by the factor) is high e.g. 0.70, then generalisability of the factor structure to the population is usually very good regardless of the sample size [37]. In the current study, the communalities of each factor was small (**0.28 – 0.77**) among all variables, thus it is recommended that a larger sample size be included in future studies with the possibility of a confirmatory factor analysis.

Another limitation relates to the convenience sample and low response rate in this study which may introduce selection bias into the results. However, a convenience sample was deemed necessary to recruit sufficient numbers of the target population. In addition, the low response rates are common when involving CALD/minority groups in health-related research and was subsequently unavoidable [38]. In addition, the use of a self-report questionnaire to examine the predictive validity of the current questionnaire is not without limitation; particularly due to the low and non-significant validity correlations of the CAPANS-PA with accelerometry measured MVPA participation [28]. The authors believe that despite the use of this seemingly imprecise measure of MVPA duration, the presented predictive correlations provide important insights into the importance of each factor and MVPA participation. It is also acknowledged that the use of certain accelerometer models can result in lower estimates of total activity due to the inability to capture cycling, water-based and upper-body based movements [39]. This may have reduced the construct validity correlations of the self-report MVPA questionnaire (CAPANS-PA) due to lower estimates of total activity. Despite the above limitations, the results of this study demonstrate that the CAPANS-C instruments provides a relatively quick, easy to complete and cost effective technique when examine psychological and social influences of PA among youth, which is ideal for large scale studies.

## Conclusion

The results of this study indicate the CAPANS-C questionnaire had acceptable reliability and evidence of predictive validity for several factors examining potential psychosocial and social correlates of PA among Chinese-Australian youth. This is the first known study to have examined the psychometric properties of a potential correlates of PA questionnaire among Chinese-Australian youth and adds to the small pool of psychometrically tested instruments both nationally and internationally.

## Competing interests

The authors declare that they have no competing interests.

## Authors’ contribution

CS directed all aspects of this study including the refinement of the CAPANS-C questionnaire, design, data collection, data entry, analysis and led the development of the manuscript. AR, KR and CB were involved in the refinement of the CAPANS-C questionnaire, conception, design and analysis framework for this study and refinement of the manuscript. AR also provided extensive support in the analyses for this study. All authors have read and approved the final manuscript.

## Pre-publication history

The pre-publication history for this paper can be accessed here:

http://www.biomedcentral.com/1471-2458/14/145/prepub
